# Combined brachytherapy and external beam radiotherapy without adjuvant androgen deprivation therapy for high-risk prostate cancer

**DOI:** 10.1186/1748-717X-9-13

**Published:** 2014-01-09

**Authors:** Toshio Ohashi, Atsunori Yorozu, Shiro Saito, Tetsuo Momma, Toru Nishiyama, Shoji Yamashita, Yutaka Shiraishi, Naoyuki Shigematsu

**Affiliations:** 1 Department of Radiology, Keio University School of Medicine, 35 Shinanomachi, Shinjuku-ku, Tokyo 160-8582, Japan; 2 Department of Radiology, National Hospital Organization Saitama Hospital, 2-1 Suwa, Wakho City, Saitama 351-0102, Japan; 3 Department of Radiology, National Hospital Organization Tokyo Medical Center, 2-5-1, Higashigaoka, Meguro-ku, Tokyo 152-8902, Japan; 4 Department of Urology, National Hospital Organization Tokyo Medical Center, 2-5-1, Higashigaoka, Meguro-ku, Tokyo 152-8902, Japan; 5 Department of Urology, National Hospital Organization Saitama Hospital, 2-1 Suwa, Wakho City, Saitama 351-0102, Japan

**Keywords:** Prostate cancer, Brachytherapy, High risk, Androgen deprivation therapy

## Abstract

**Background:**

To report the outcomes of patients treated with combined iodine-125 (I-125) brachytherapy and external beam radiotherapy (EBRT) for high-risk prostate cancer.

**Methods:**

Between 2003 and 2009, I-125 permanent prostate brachytherapy plus EBRT was performed for 206 patients with high-risk prostate cancer. High-risk patients had prostate-specific antigen ≥ 20 ng/mL, and/or Gleason score ≥ 8, and/or Stage ≥ T3. One hundred and one patients (49.0%) received neoadjuvant androgen deprivation therapy (ADT) but none were given adjuvant ADT. Biochemical failure-free survival (BFFS) was determined using the Phoenix definition.

**Results:**

The 5-year actuarial BFFS rate was 84.8%. The 5-year cause-specific survival and overall survival rates were 98.7% and 97.6%, respectively. There were 8 deaths (3.9%), of which 2 were due to prostate cancer. On multivariate analysis, positive biopsy core rates and the number of high-risk factors were independent predictors of BFFS. The 5-year BFFS rates for patients in the positive biopsy core rate <50% and ≥50% groups were 89.3% and 78.2%, respectively (*p* = 0.03). The 5-year BFFS rate for patients with the any single high-risk factor was 86.1%, compared with 73.6% for those with any 2 or all 3 high-risk factors (*p* = 0.03). Neoadjuvant ADT did not impact the 5-year BFFS.

**Conclusions:**

At a median follow-up of 60 months, high-risk prostate cancer patients undergoing combined I-125 brachytherapy and EBRT without adjuvant ADT have a high probability of achieving 5-year BFFS.

## Background

The prognosis for men with clinically localized, high-risk prostate cancer treated with external beam radiotherapy (EBRT) has improved significantly over the last 15 years [1-7]. Most notably, the addition of androgen deprivation therapy (ADT) to standard dose EBRT has been shown in several large, randomized studies to increase cause-specific survival (CSS) and overall survival (OS) [1,2]. In addition, increasing the external beam dose to 78–80 Gy has led to improvements in biochemical failure-free survival (BFFS) [3-6]. However, even with these improvements, high-risk prostate cancer remains a therapeutic challenge for both urologists and radiation oncologists.

Stock et al. documented results for a series of patients with high-risk disease receiving trimodality therapy consisting of brachytherapy, EBRT and ADT, reporting excellent biochemical and pathologically confirmed local control [8]. Their group also reported a recent series showing favorable distant control and disease-specific survival in men with Gleason score 8–10 disease [9], and long-term biochemical control in men with extra-prostatic disease [10]. Brachytherapy provides a means to further raise the local dose and has been used in an attempt to improve results in men with high-risk disease. With high biologic effective doses (BED) being achievable with EBRT plus brachytherapy, BFFS rates of 85–90% have reportedly been obtained in large cohorts of men with high-risk disease [11,12]. Some previous studies demonstrated a benefit of ADT used in conjunction with EBRT to treat locally advanced prostate cancer [1,2,13]. However, these studies, which demonstrated an advantage with the addition of ADT, were conducted during a period when radiation doses may have been inadequate to control all local disease. Clear evidence for using adjuvant ADT when much higher radiation doses are delivered is thus lacking.

The use of permanent prostate brachytherapy employing iodine-125 (I-125) seeds has expanded rapidly in Japan since the establishment of guidelines for this treatment modality and revision of the dosimetric regulations related to radiation hazards and safety in 2003. In this report, we summarize the clinical outcomes of patients in our experience receiving combined therapy consisting of permanent prostate brachytherapy and EBRT without adjuvant ADT.

## Methods

Between September 2003 and June 2009, 206 consecutive Japanese patients with high-risk localized prostate cancer were treated with combined modality therapy consisting of I-125 permanent seed implantation and supplemental EBRT at either the National Hospital Organization Tokyo Medical Center or the National Hospital Organization Saitama Hospital. These patients included men with a prostate-specific antigen (PSA) level higher than 20 ng/mL, and/or Gleason score ≥ 8, and/or Stage T3. Clinical T stage was classified by combination of magnetic resonance imaging finding and digital examination by urologist. There were no treatment policy discrepancies between the National Hospital Organization Tokyo Medical Center and National Hospital Organization Saitama Hospital. One hundred and one patients (49.0%) received neoadjuvant ADT with the aim of prostate volume reduction or a longer waiting time. Regarding the aim of volume reduction, patients with prostate volumes >40 cc usually underwent ADT because Japanese national policy for patient discharge criteria mandates that total seed activity be kept below 1,300 MBq. None of our present patients received adjuvant ADT. ADT consisted of luteinizing hormone-releasing hormone agonist alone or in combination with an anti-androgen. The length of ADT duration was decided at the discretion of the treating urologist, and the median duration of ADT was 4 months (range, 3–86 months). This retrospective study was approved by the each hospital’s local Institutional Review Board.

The implant technique and dose constraints were previously described in detail [14-16]. Early in the study period, the preplanning method was used in the first 25 patients, and from December 2004 onward, the procedure was changed to the real-time planning method. All procedures were conducted utilizing I-125 free seeds, being the only approved radioisotope available for permanent prostate brachytherapy in Japan. The prescribed minimum peripheral doses were 100 Gy in the preplanning method era and 110 Gy in the real-time planning method era, respectively. Post-implant dosimetry was performed 1 month after implantation, and the minimal dose received by 90% of the prostate (prostate D90) was the post-implant variable analyzed.

Supplemental EBRT was delivered 4 to 8 weeks after implantation. In general, EBRT consisted of a median dose of 45 Gy (range, 28.8–50.4 Gy) delivered in 1.8 Gy fractions using 6–10 MV photons delivered via a three-dimensional conformal technique. For all patients, the target volume consisted of the prostate gland and seminal vesicles. The BED was calculated from the prostate D90 and the EBRT dose using an α/β ratio of 2 (Gy2), applying the formulas described previously by Stock et al. [17]. The total BED for the combination therapy was the sum of the BED from the implant and that from the EBRT.

Planned follow-up was by PSA blood tests and physical examination every 3 months for the first 2 years, every 6 months thereafter. The primary outcome measure was BFFS. Biochemical failure was determined using the nadir +2 ng/mL definition (the Phoenix definition). Patients meeting the criteria for biochemical failure but showing a subsequent decrease to <0.5 ng/mL without intervention were classified as having a benign bounce, and were excluded from the analysis of failure. Acute toxicity was considered to be symptoms developing within the first year after implantation. Late toxicity was defined as any symptom developing after the first year, or symptoms that developed during the first year and persisted ≥12 months. Toxicity was scored by the Common Terminology Criteria for Adverse Events version 4.0.

Actuarial survival curves were calculated by the Kaplan-Meier method to determine BFFS, CSS, and OS. Multivariate Cox regression analysis including age, PSA level, Gleason score, positive biopsy core rates, number of high-risk factors, neoadjuvant ADT, prostate D90, and BED was conducted to test for predictors of BFFS. Analyses were carried out using SPSS 20.0 (SPSS Inc., Chicago, IL, USA). All tests were two-sided, and statistical significance was set at *p* < 0.05.

## Results

Clinical, treatment and dosimetric parameters for the 206 patients included in the analysis are detailed in Table 1. The median follow-up time was 60 months (range, 9–112 months).

**Table 1:** Clinical, treatment and dosimetric parameters

	**Median (range)**	**Count (%)**
Continuous variables		
Age (years)	70 (54–86)	
Initial PSA (ng/mL)	11.95 (3.7–48.0)	
Positive biopsy rate (%)	33.0 (13.0–100)	
Prostate D90 (Gy)	124.8 (100.0–206.5)	
BED (Gy2)	213.5 (178.5–245.5)	
Categorical variables		
PSA level in ng/mL		
<10		86 (41.7%)
10-20		39 (19.0%)
≥ 20		81 (39.3%)
Gleason score		
5-6		26 (12.6%)
7		54 (26.2%)
8-10		126 (61.2%)
Clinical T stage		
T1-T2a		142 (69.0%)
T2b-T2c		46 (22.4%)
T3a		18 (8.6%)
No. of high-risk features		
1		186 (90.3%)
2		19 (9.2%)
3		1 (0.5%)
Neoadjuvant ADT		
Yes		101 (49.0%)

Abbreviations: PSA = prostate specific antigen; D90 = the minimal dose received by 90% of the prostate; BED = biologically effective dose; ADT = androgen deprivation therapy.

**Figure 1:**
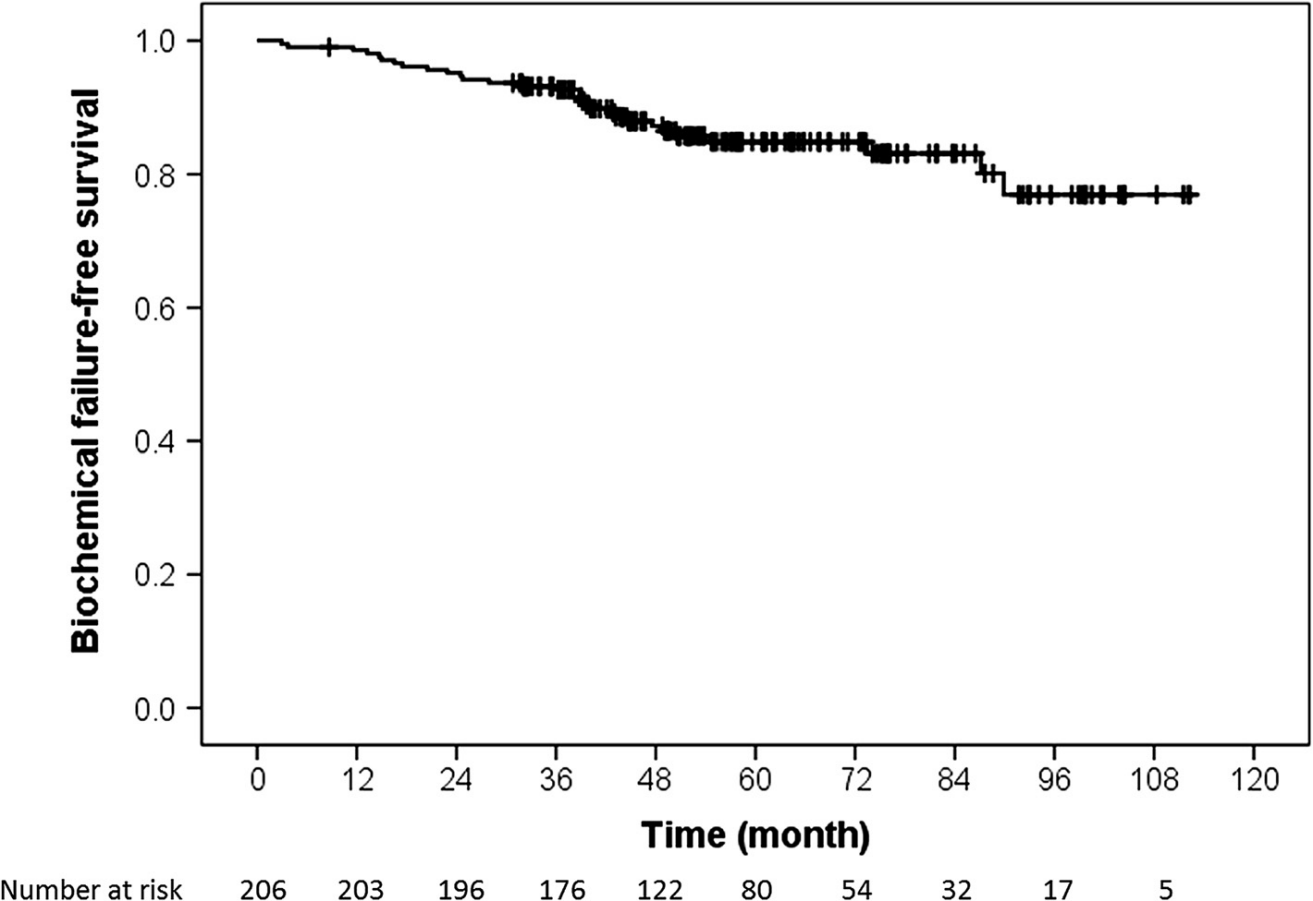
Kaplan-Meier biochemical failure-free survival curve for the 206 patients in the high-risk study population.

Of the 206 patients, 30 developed PSA failure, yielding an actuarial BFFS rate of 84.8% at 5 years (Figure 1). The median time to biochemical failure was 37.2 months in those who failed. Of the 30 patients with PSA failure, 20 underwent post-treatment biopsy. Five of these 20 patients had pathologically-proven local recurrence. The patterns of clinical failure were local recurrence in 4 patients, distant metastases in 13, and both in 1. There were 8 deaths (3.9%), of which 2 were due to prostate cancer. The 5-year CSS for the entire cohort was 98.7%. The 5-year OS for the entire cohort was 97.6%.

Acute grade 2 gastrointestinal (GI) and genitourinary (GU) toxicity was experienced by 12 patients (5.8%) and 20 patients (14.5%), respectively. Late grade 2 GI and GU toxicity was experienced by 18 patients (8.8%) and 21 patients (10.2%), respectively. None of the patients experienced Grade ≥3 acute or late toxicity. The late grade 2 GI toxicities primarily related to rectal bleeding and the late grade 2 GU toxicities consisted of urinary urgency or retention. Rectal or urethral doses were not associated with the development of grade 2 GI or GU toxicity on univariate analysis.

**Figure 2:**
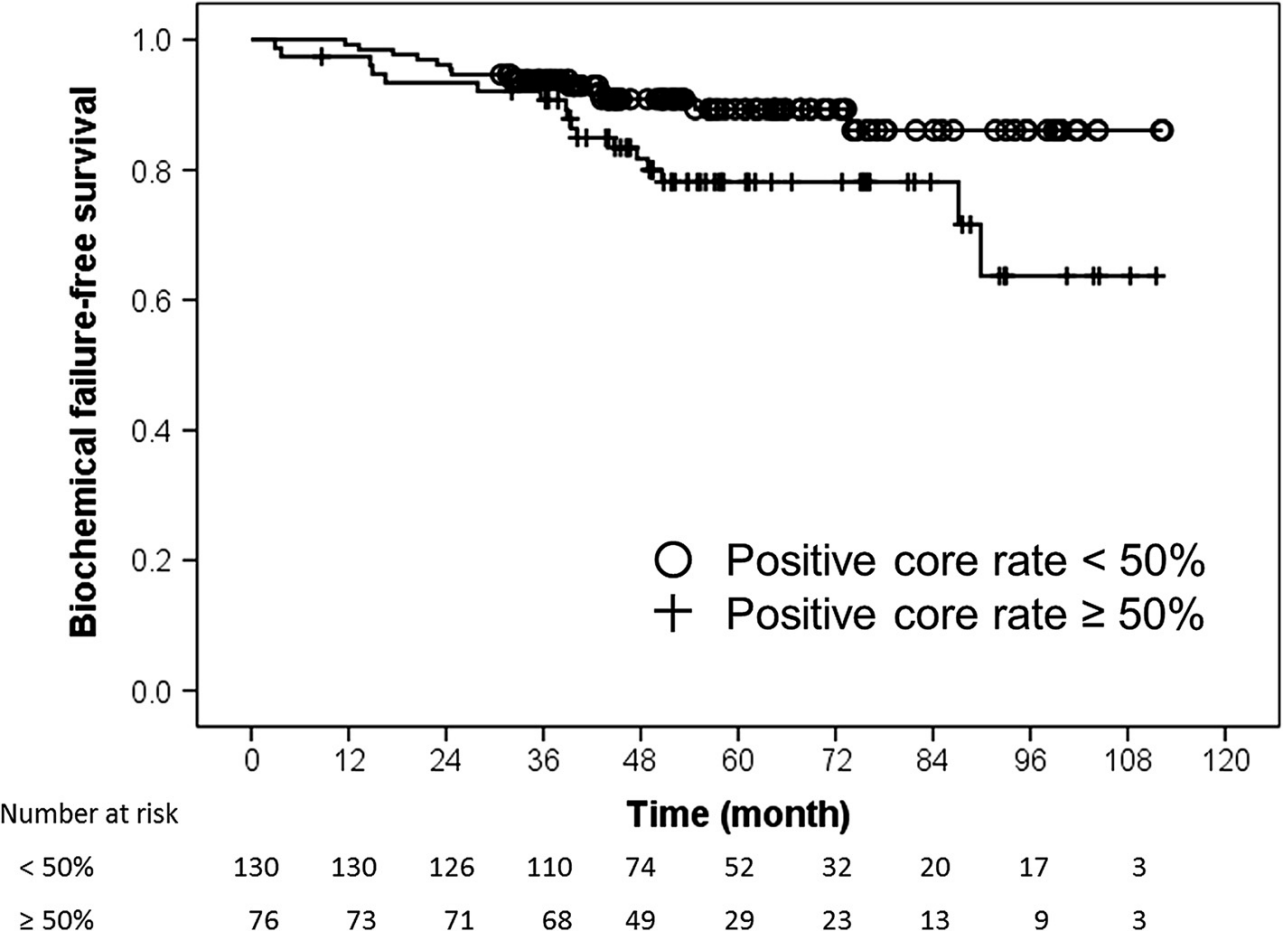
**Kaplan-Meier biochemical failure-free survival curves as a function of positive biopsy core rates.** Open circles indicate the time of last follow-up for the biochemical failure-free patients with a positive core rate <50% (n = 130). Plus symbols correspond to censored patients with a positive core rate ≥50% (n = 76).

**Figure 3:**
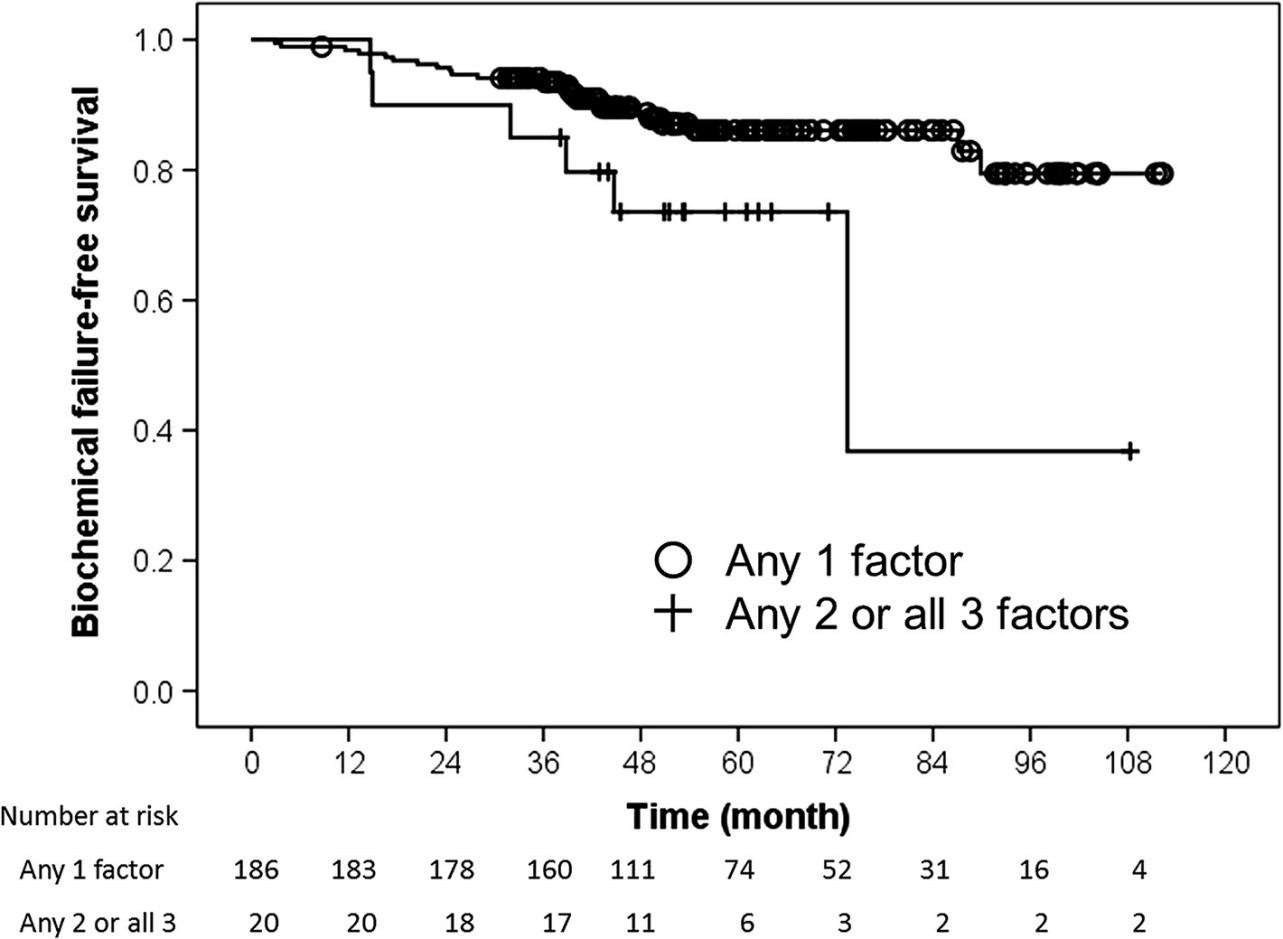
**Kaplan-Meier biochemical failure-free survival curves as a function of the number of high-risk factors.** Open circles indicate the time of last follow-up for the biochemical failure-free patients with any single high-risk factor (n = 186). Plus symbols correspond to censored patients with any 2 or all 3 high-risk factors (n = 20).

On multivariate Cox regression analysis, positive biopsy core rates and number of high-risk factors were independent predictors of BFFS by the Phoenix definition (Table 2). The positive biopsy core rates were divided into subgroups: <50% (n = 130, 63.1%) and ≥50% (n = 76, 36.9%). As shown in Figure 2, the 5-year BFFS rates for patients in the positive biopsy core rate <50% and ≥50% groups were 89.3% and 78.2%, respectively (*p* = 0.03). Figure 3 shows BFFS stratified by numbers of high-risk factors (any 1 vs. any 2 or all 3). The 5-year BFFS rates for patients with any single high-risk factor was 86.1%, compared with 73.6% for those with any 2 or all 3 high-risk factors (*p* = 0.03). Neoadjuvant ADT did not improve the 5-year BFFS (87.1% vs. 82.1%, *p* = 0.11), according to analysis employing the log-rank test.

**Table 2:** Cox regression for biochemical freedom from failure

			**95% CI**	
**Variable**	**Significance (*p *** **value)**	**Hazard rate**	**Lower**	**Upper**
Age	0.936	0.998	0.940	1.061
PSA level	0.313	0.710	0.365	1.381
Gleason score	0.850	1.193	0.190	7.495
Positive biopsy rate	0.009*	2.940	1.314	6.577
No. of high-risk features	0.023*	4.162	1.218	14.220
Neoadjuvant ADT	0.064	0.468	0.946	1.045
Prostate D90	0.515	1.015	0.971	1.061
BED	0.833	0.995	0.946	1.046

Abbreviations: CI = confidential interval; PSA = prostate specific antigen; ADT = androgen deprivation therapy; prostate D90 = the minimal dose received by 90% of the prostate; BED = biologically effective dose.

*p < 0.05.

## Discussion

Although some patients with high-risk factors may have subclinical distant metastatic disease at diagnosis, prior trials reported improved BFFS for patients with high-risk prostate cancer who received higher doses of EBRT [4-6]. This finding refutes the hypothesis that most patients with high-risk factors have subclinical distant metastases at diagnosis, but rather, supports an aggressive loco-regional treatment approach. In addition, Do et al. reported a 5-year biochemical progression-free survival rate of 20% for patients with Gleason scores of 8–10 who were treated with radical prostatectomy and a rate of 30% for those given conventional doses of EBRT [18]. However, 65% of patients undergoing prostatectomy with adjuvant EBRT were biochemically free of disease at 5 years. This study also supports an aggressive loco-regional treatment approach.

Brachytherapy provides a means to further escalate the local dose and has been used in an effort to improve results in men with high-risk disease [11,12]. Stone et al. reported a multicenter cohort study of 3,928 brachytherapy patients with a median follow-up of 42 months [11]. For the cohort as a whole, the respective BFFS rates for low-, intermediate-, and high-risk patients were 84%, 77%, and 64%. However, the 1,100 men who received a higher BED of >200 Gy via their implant, with or without EBRT, had much more favorable outcomes. Among these men, BFFS for low-, intermediate-, and high-risk patients were 88%, 94%, and 90%, respectively. The range seen in BED values is mostly due to inherent inaccuracies in the implant procedure itself, with the resulting variation in dosimetry developing after implantation. The EBRT dose variation stems mainly from the policy of adjusting these doses based on the final dosimetric outcome of the implant. As a result of these adjustments, the combination of brachytherapy and EBRT resulted in very high BED (median 213.5 Gy2, range 178.5–245.5 Gy2) in our study. This dose is much higher than the 81 Gy (associated BED of 154 Gy2) commonly prescribed for intensity-modulated radiotherapy.

High-dose-rate (HDR) brachytherapy is also a technique that allows the delivery of very high BED. Martinez et al. performed a dose escalation investigation starting with HDR doses of 5.5 Gy × 3 and ending with 11.5 Gy × 2 for the HDR component of treatment [19]. The mean dose of EBRT was 46 Gy. They found that when the BED (α/β ratio of 1.2) was ≥ 268 Gy there was less biochemical failure, better local control, and fewer cases of distant metastasis. Kotecha et al. reported on the outcomes of 229 patients with clinically localized prostate cancer treated with a HDR brachytherapy boost (5.5 Gy × 3 to 7.5 Gy × 3) followed by EBRT (most patients were treated to 50.4 Gy) and found that a higher BED (> 190 Gy, α/β ratio of 2) resulted in improved BFFS and distant metastases free survival in high-risk patients [20].

In our present study, the prognostic significance of BFFS was investigated in high-risk prostate cancer patients and we found positive biopsy core rates and the number of high-risk factors to be independent predictors of BFFS. The positive biopsy core rates as determined by transrectal ultrasound-guided biopsy have been suggested as potential prognostic factors for enhancing the standard risk stratification for prostate cancer patients treated with EBRT [21-23]. Huang et al. analyzed 1,056 patients who were treated with modern EBRT techniques and found the positive biopsy core rate to be an independent predictor of highly relevant clinical outcomes. The association of the positive biopsy core rate with distant metastasis was especially robust and was unaffected by the use of either hormone therapy or high-dose EBRT [22].

The relationship between the positive biopsy core rate and BFFS has been examined in studies of patients treated with brachytherapy [24-26]. Kestin et al. reported on 190 men treated with a combination of EBRT and high-dose rate brachytherapy [24]. On multivariate analysis, the positive biopsy core rate was associated with BFFS and the development of clinical recurrence. Moreover, Merrick et al. reported on 255 men treated with seed implantation with or without EBRT. On multivariate analysis, the positive biopsy core rate and pretreatment PSA level were the only significant predictors of BFFS [25]. When low, intermediate, and high-risk patients were stratified by the positive biopsy core rate, a non-significant trend for increased biochemical recurrence was observed as positive biopsy core rates rose. The number of recurrences in their patient population was quite low, and it is possible that their study is underpowered to show a clinically significant effect of positive biopsy core rates after stratification by risk group. Rossi et al. described the 5-year estimate of the BFFS rate as being 95% for patients with a less than 50% positive biopsy core rate versus 63% in those with a rate of more than 50% [26]. These reports support the results of our present study, but the potential value of positive biopsy core rates for predicting CSS and OS requires longer follow-up and could not be quantified in our present study.

Several retrospective studies have assessed the associations of the number of high-risk factors and clinical outcomes of men given brachytherapy-based treatment [27,28]. Wattson et al. analyzed the impact of the number of high-risk factors on prostate cancer-specific mortality (PCSM) [27]. The adjusted hazard ratio for PCSM for those with at least two high-risk factors (as compared with one) was 4.8 (95% confidence interval, 2.8–8.0; *p* < 0.001). When the high-risk factors were analyzed separately, Gleason score 8–10 was most significantly associated with increased PCSM. Several studies have reported similar findings for men treated with definitive EBRT alone or EBRT plus hormone therapy, and for men undergoing radical prostatectomy [29-31].

Our study showed that clinicians may base treatment selection decisions on the number of high-risk factors and positive biopsy core rates, and found that men with more high-risk factors and a positive biopsy core rate ≥50% were likely to be selected for intensified treatments such as trimodality therapy including brachytherapy, EBRT and ADT. However, the results of this study cannot be used to conclude that brachytherapy-based trimodality therapy necessarily leads to improved rates of control and survival as compared with alternative treatments that do not include brachytherapy, such as radical prostatectomy or definitive EBRT with or without ADT.

The addition of ADT to standard dose EBRT was a significant breakthrough for men with high-risk disease and has resulted in major improvements in prostate cancer-specific survival and OS [1,2]. Although ADT has been studied in only a few trials with brachytherapy, in one study by Merrick et al. [32], ADT improved the 10-year BFFS rate when added to the combination of brachytherapy and EBRT versus combined therapy alone for high-risk prostate cancer. Meanwhile, according to the retrospective review by Lee et al. 80% of high-risk hormone-naive patients with a high-quality implant remained free of biochemical failure at 5 years [33]. In our study, there were too few patients with prostate D90 <110 Gy to obtain a dose–response curve and the results of Lee et al. are identical to our 84.8% BFFS rate at 5 years. These results in hormone-naive patients further substantiate the importance of aggressive loco-regional treatment in securing long-lasting biochemical control in high-risk patients. A clinical randomized trial has been conducted to investigate the efficacy of adjuvant ADT following the combination of brachytherapy and EBRT for high-risk prostate cancer patients in Japan [34].

The limitations of this study are that the median follow-up is only 60 months and it was retrospective. Neoadjuvant ADT was administered at the discretion of the treating urologist for reasons including prostate volume reduction or to achieve a longer waiting time until seed implantation, therefore the duration of neoadjuvant ADT was not controlled, though all other treatments were uniform in most patients.

## Conclusions

At a median follow-up of 60 months, high-risk prostate cancer patients who underwent combined I-125 brachytherapy and EBRT without adjuvant ADT have a high probability of achieving 5-year BFFS. Positive biopsy core rates and the number of high-risk factors significantly impact BFFS. Additional follow-up is mandatory to determine the durability of these results.

## Abbreviations

EBRT: External beam radiotherapy; ADT: Androgen deprivation therapy; BFFS: Biochemical failure-free survival; CSS: Cause-specific survival; OS: Overall survival; BED: Biologic effective doses; I-125: Iodine-125; PSA: Prostate-specific antigen; Prostate D90: Minimal dose received by 90% of the prostate; GI: Gastrointestinal; GU: Genitourinary; HDR: High-dose-rate; PCSM: Prostate cancer-specific mortality.

## Competing interests

The authors declare that they have no competing interests.

## Authors’ contributions

TO and AY collected the data, interpreted the results, and performed the statistical analysis. SS, TM, TN and SY participated in data acquisition and helped to analyze the data. YS and NS contributed to data analysis. All authors read and approved the manuscript.
